# Severe constipation as the first clinical manifestation in multiple endocrine neoplasia type 2B: a case report and literature review

**DOI:** 10.1186/s12887-020-02224-4

**Published:** 2020-06-29

**Authors:** Lidan Zhang, Yan Guo, Lei Ye, Wenli Lu, Zhiya Dong, Wei Wang, Yuan Xiao

**Affiliations:** 1 Department of Pediatrics, Ruijin Hospital, Shanghai Jiao Tong University School of Medicine, Shanghai, 200025 China; 2 Department of Endocrine and Metabolism, Ruijin Hospital, Shanghai Jiao Tong University School of Medicine, Shanghai, 200025 China

**Keywords:** Multiple endocrine neoplasia 2B, Medullary thyroid carcinoma, Multiple mucosal neuroma, Hirschsprung disease, Proto-oncogene

## Abstract

**Background:**

The occurrence of multiple endocrine neoplasia type 2B (MEN2B) in Asians is very rare. In particular, patients with intractable constipation as the main clinical manifestation are even rarer. Atypical clinical manifestations are likely to lead to a diagnostic delay. In this report, we described a case of a delayed diagnosis of MEN2B, and the first clinical manifestation was intractable constipation.

**Case presentation:**

A female teenager had suffered from intractable constipation since infancy. Because the colonoscopy and biopsy results from local hospitals did not confirm the presence of congenital megacolon, the girl had been followed up at a local clinic for a long time. The diagnosis was not confirmed until thyroid masses were found in the Pediatric Department of Shanghai Ruijin Hospital when she was 12 years old. According to our detailed evaluation, she suffered from Hirschsprung disease (HD), growth retardation, medullary thyroid carcinoma (MTC) and mucosal neuroma due to a mutation in the *RET* gene. Thus, the diagnosis of MEN2B was confirmed. Afterward, the girl underwent several surgeries and was still being followed up before the article was published.

**Conclusion:**

MEN2B has atypical clinical symptoms in the early stage. Refractory constipation may be the only clinical manifestation that lasts for several years. Therefore, we recommend that early screening and gene sequencing should be performed for patients with severe constipation due to HD to determine the cause of the disease and to improve the survival outcome.

## Background

Multiple endocrine neoplasia (MEN) is a general term for two or more endocrine glands with tumor lesions. According to its clinical manifestations and types of mutation, this condition can be divided into MEN1 (Online Mendelian Inheritance in Man (OMIM) number: 131100), MEN2 and familial medullary thyroid carcinoma (FMTC) (OMIM number: 155240). Furthermore, MEN2 also has two subtypes: MEN2A (OMIM number: 171400) and MEN2B (OMIM number: 162300). MEN2B is a rare autosomal dominant genetic disease caused by a mutation of the *RET* proto-oncogene. The main clinical manifestations are medullary thyroid carcinoma (MTC), multiple mucosal neuroma, pheochromocytoma (PHEO), and Marfan-like habitus [1]. The overall incidence of MEN2B is approximately 1:35,000 ~ 1:40,000 [2], and it only accounts for 5–10% of all cases of MEN2 [3]; MEN2B was officially named by Chong [4] in 1975. To date, only 20 cases of MEN2B have been reported in mainland China, and few cases of children have been documented.

Patients with MEN2B often have gastrointestinal (GI) symptoms such as abdominal pain, constipation or diarrhea due to Hirschsprung disease (HD), which usually occurs during childhood and early adulthood [5, 6]. Approximately 50% of the patients in China had gastrointestinal tract involvement, and such conditions were noted in 61–90% of patients with different ethnicities [7, 8]. However, the non-MTC clinical manifestations were often overlooked, leading to misdiagnosis. The average age of diagnosis of MEN2B is 14.2 years old [8].

Here, we report a case of a Chinese girl with MEN2B who had severe constipation since infancy due to a pathogenic mutation in *RET*.

## Case presentation

### Clinical data

A 12-year-old girl born in the Zhejiang Province of China was admitted to the Department of Pediatrics, Ruijin Hospital, Shanghai Jiao Tong University School of Medicine in February 2016 with chief complaints of constipation for 12 years and neck lumps for 2 years. She was the first-born child to her parents and was born at 38 weeks and 4 days of gestation after an unremarkable pregnancy. No obvious abnormalities were found during her neonatal period. She had experienced constipation since infancy. The patient had no family history of MTC or HD. Nevertheless, she had a younger half-brother with pancreatic dysplasia with whom she shared a father. She underwent colonoscopy in a local tertiary hospital due to her intractable constipation when she was 2 years old. However, the diagnosis of congenital megacolon could not be confirmed according to the results of the intestinal biopsy. Afterward, she was followed up at a clinic. Two masses were found in her neck 2 years ago and have gradually increased in size since being identified.

**Fig. 1:**
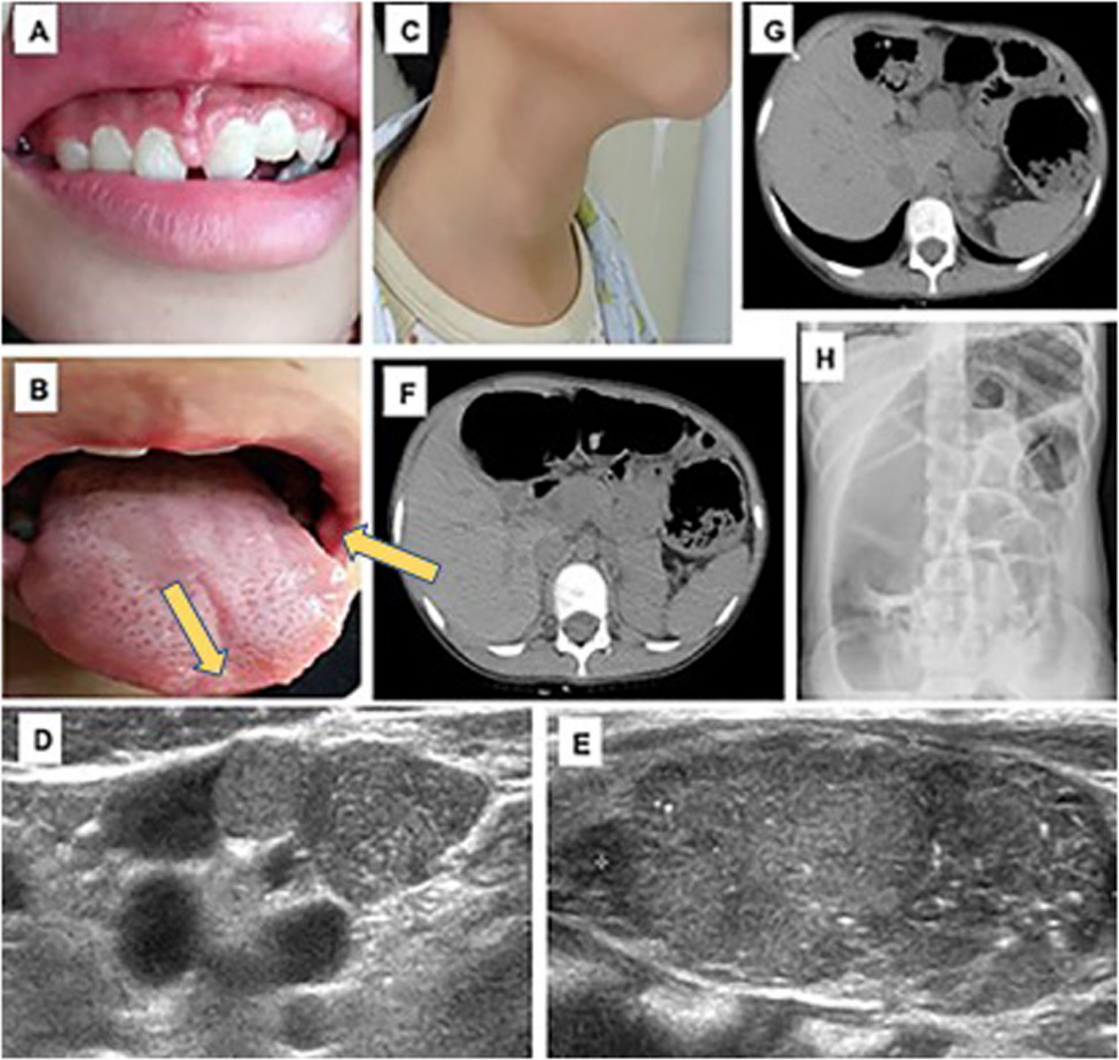
Clinical Characteristics of the Patient (**a**) Thickened lips. (**b**) Neuromas affecting the gingival tissue, tongue and oral mucosa (as yellow arrows indiated). (**c**) Thyroid nodule. (**d**) Multiple metastatic lymph nodes .(**e**) Thyroid enlargement (with multiple calcifications). (**f**)(**g**) Colon dilatation.(**h**) Colon wall thickening

On the examination, her weight was 26.8 kg (− 2.08 SD), her height was 136 cm (− 2.56 SD) and she had a BMI of 14.49 kg/m^2^ (− 1.57 SD). She had not exhibit Marfan-like habitus. Her external genitalia and breasts were Tanner stage I, and she had no pubic hair. Therefore, she had not yet undergone puberty. Her blood pressure was 100/64 mmHg. The physical examination also revealed multiple painless, firm nodules on her gingival tissue, tongue and buccal mucosa, and these were considered mucosal neuromas (Fig. 1 a, b). Her thyroid was enlarged, and multiple hard nodules could be palpable on the surface of the thyroid (Fig. 1 c). There was no palpable mass in her abdomen.

Her serum levels of free triiodothyronine (FT3), free tetraiodothyronine (FT4) and thyroid-stimulating hormone (TSH) were normal, but her calcitonin level was very high (1187 pg/ml), and her parathyroid hormone (PTH) level was slightly elevated (Table 1). Moreover, her serum carcinoembryonic antigen (CEA) level was much higher than normal (655.4 ng/ml). The level of insulin-like growth factor 1 level (61 ng/ml) was lower than the normal reference according to her age. Her other hormone (serum insulin, growth hormone, prolactin, cortisol, adrenaline, norepinephrine) level and electrolyte levels were normal (Table 1).

**Table 1:** Main results of laboratory investigations on the patient before the first surgery

Variable	Unit	Reference Range	On admission
TSH	uIU/ml	0.35–4.94	3.09
FT3	pmol/l	2.63–5.70	5.24
FT4	pmol/l	9.01–19.04	13.85
T3	nmol/l	0.89–2.40	1.80
T4	nmol/l	62.6–150.8	94.11
Calcitonin	pg/ml	< 10	> 1187
PTH	pg/ml	15.0–68.3	75.5
CEA	ng/ml	< 5.0	655.4
AFP	ng/ml	0–8.78	2.92
Ca	mol/l	2.0–2.75	2.29
P	mol/l	0.8–1.6	1.62
E	pg/ml	14–90	57.4
NE	pg/ml	19–121	94.4
IGF-1	ng/ml	385–665	61

Remark: *TSH* Thyroid stimulating hormone, *FT3* Free triiodothyronine, *FT4* Free tetraiodothyronine, *T3* Triiodothyronine, *T4* Tetraiodothyronine, *PTH* Parathyroid hormone, *CEA* Carcinoembryonic antigen, *AFP* Alpha-fetoprotein, *P* Phosphorus, *E* Epinephrine, *NE* Norepinephrine, *IGF-1* Insulin-like growth factors −1

Two masses were found in each lobe of her thyroid along with multiple metastatic lymph nodes by ultrasound (Fig. 1 d). The size of the right mass was 37 mm × 20 mm and that of the left mass was 43 mm × 20 mm (Fig. 1 e). Both masses were classified as Thyroid Imaging Reporting and Data System (TI-RADS) 5. The abdominal X-ray and CT scan revealed transverse colonic dilatation and colonic wall thickening (Fig. 1 f, g, h), which suggested a diagnosis of megacolon. There were no obvious abnormalities in her head MRI or adrenal CT scan.

**Fig. 2:**
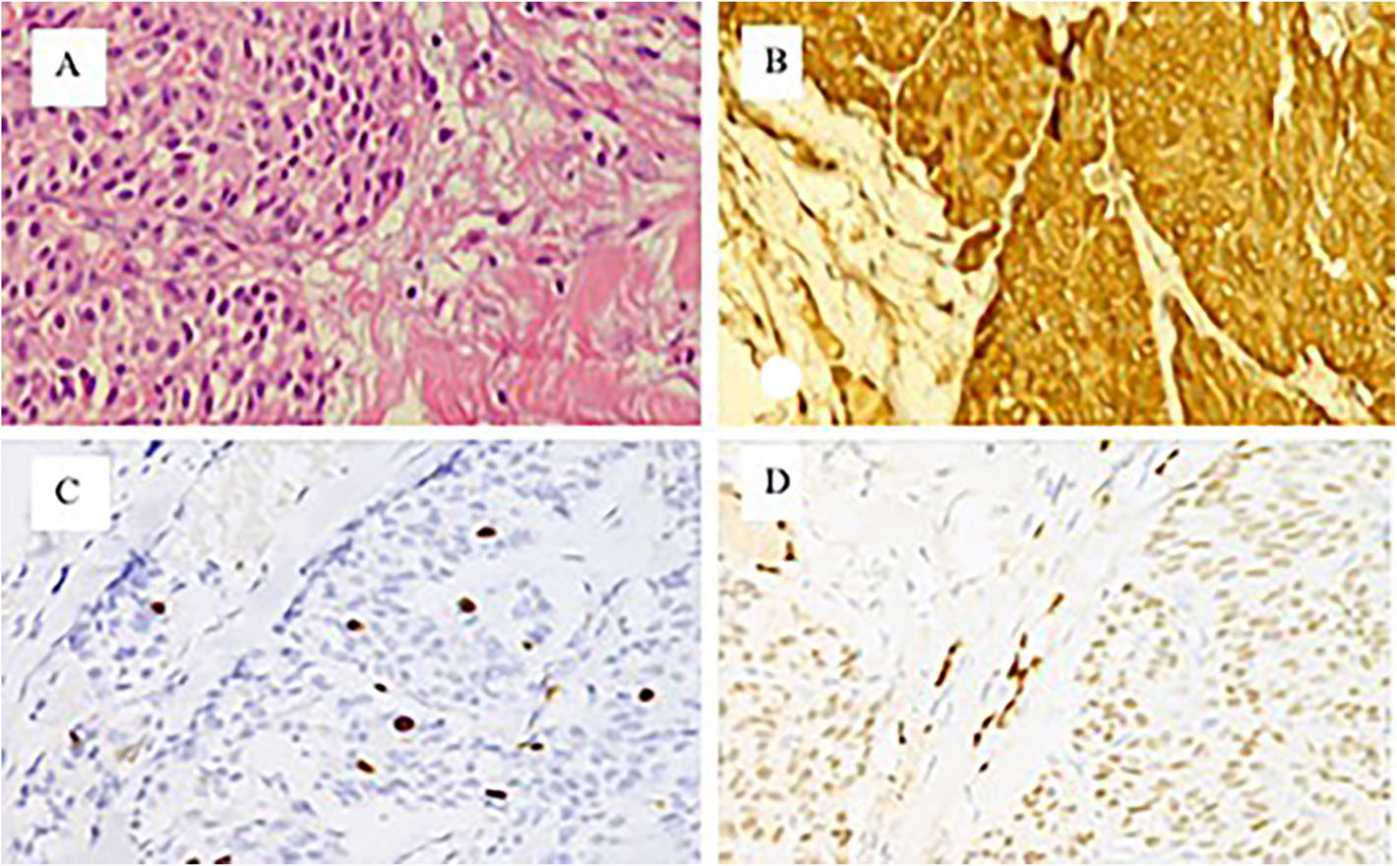
Pathological image of the thyroid tumor (**a**) Pathological image of the thyroid tumor (HE staining, 40 × 10), showing the arrangement of spindle-shaped medullary carcinoma cells and amyloid deposition; (**b**)(**c**)(**d**) are immunohistochemical photomicrographs (DAB staining, 40 × 10) that suggest calcitonin (+), Ki67 (+), and TTF-1 (+)

She underwent an extended radical thyroidectomy and cervical lymph node dissection for thyroid cancer. As expected, the postoperative pathological diagnosis changed to bilateral MTC (T3N1bM0 (stage IV A)), with 24/38 metastases observed in the lymph nodes (Fig. 2a, b, c, d).

### Genetic test

**Fig. 3:**
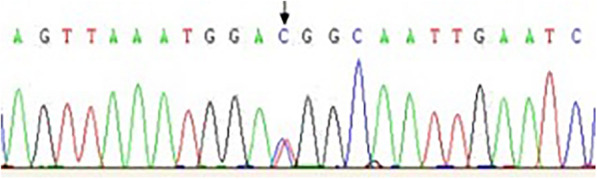
RET gene mutation site sequencing map (c. T2753C, p. M918T) of the patient

A pathogenic mutation in the *RET* gene (c.2753 T > C, p. M918T) was found in the proband but not in her father or in her younger half-brother (Fig. 3). Since her mother’s DNA was not obtained, it was impossible to determine whether the mutation was de novo. However, this is a hot spot variation in *RET,* and it has been proven to be a causative mutation of MEN2B. According to the American College of Medical Genetics and Genomics (ACMG) guidelines, this mutation was defined as a pathogenic variation [9]. Finally, a diagnosis of MEN2B was confirmed by phenotyping and genotyping.

### Follow-up

After the operation, the patient was given L-thyroxine 50 μg/d as substitution therapy. The dosage was adjusted according to her thyroid function, which was reviewed regularly in our pediatric endocrinology clinic.

Her calcitonin level decreased gradually from 1187 pg/ml to 850.8 pg/ml. The calcitonin level increased again and reached 1072.1 pg/ml 5 months after the first operation. Then, she was arranged to undergo another CT scan. Afterward, cervical lymph node and mediastinal metastases of the MTC were confirmed. In September 2016, she underwent mediastinal tumor resection and extended radical surgery for thyroid cancer. Since this surgery, the serum levels of calcitonin and CEA have fluctuated within 442.5 pg/ml ~ 2000 pg/ml and 9.95 ng/ml ~ 98.32 ng/ml, respectively.

In October 2017, the child underwent colectomy in another hospital because of severe constipation induced by megacolon. She was diagnosed with HD since the pathology of colon indicated ganglioneuromatosis in the whole colon.

**Fig. 4:**
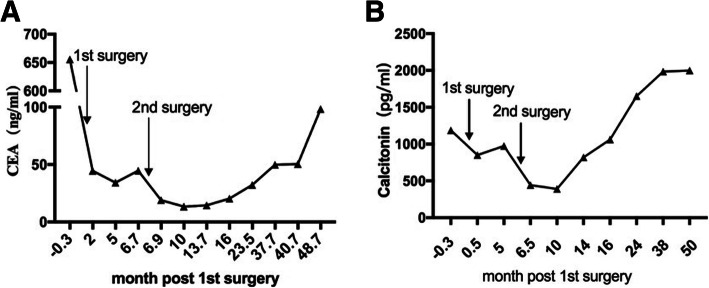
**a** Follow-up of the CEA index **b** Follow-up of the calcitonin index

Since then, she has achieved catch-up growth and has been followed in our pediatric clinic for monitoring of her growth and development, IGF-1 level, blood pressure, thyroid function, adrenal function, and levels of CEA and calcitonin (Fig. 4a, b). She began puberty after the MTC was removed and menstruated 1 year ago. During the most recent follow-up, she was 156.8 cm (− 0.62 SD) tall.

## Discussion and conclusions

The clinical symptoms of MEN2B include mucosal neuroma, Marfan-like habitus, thyroid carcinoma (especially MTC), PHEO, gastrointestinal symptoms, and specialized facial features. MEN2B combined with GI symptoms mainly includes abdominal pain, constipation or diarrhea, and often includes megacolon disease. Megacolon symptoms usually occur in childhood and early adulthood [10]. Approximately 50% of Chinese patients have gastrointestinal symptoms, while this prevalence in other races is approximately 61–90% [7, 8]. However, the non-MTC clinical manifestations are often overlooked, leading to a delayed diagnosis. The average age of diagnosis of MEN2B is 14.2 years [8]. In this case, the patient had got intractable constipation since early infancy. She was not diagnosed until a neck mass was found. However, lymph node metastasis and thymus metastasis occurred when MTC was diagnosed, and the best surgical opportunity had been missed. It has been suggested that *RET* gene sequencing should be performed to rule out MEN2B when HD is considered [11]. The curative rate of early thyroidectomy for MTC without metastasis can reach as high as 100%. Since the prevalence of MTC is 100% in patients with the p.M918T mutation of the *RET* gene, the current guidelines of the American Thyroid Association (ATA) recommend that if children have this mutation, thyroidectomy be performed before the patients reach 1 year in age to prevent MTC in the future [2]. Thus, the early diagnosis of MEN2B and *RET* gene sequencing are important to improving the prognosis of suspected children without a familial history of MTC. It is useful to perform *RET* proto-oncogene detection in infants with HD, and this analysis may find new sporadic patients with MEN2 [12]. Additionally, mucosal neuromas and intestinal ganglioneuromas are characteristic signs and pathological findings of MEN2B, respectively, and can contribute to screening for MEN2B. In our case, the girl developed mucosal neuromas on her tongue, lip and buccal mucosa. If these characteristics had been recognized by local doctors, she might have undergone thyroidectomy much earlier than she did.

In the PubMed database, 927 articles were related to MEN2B, of which 261 cases were of patients aged 0–18 years old. In these articles, the clinical manifestations of MEN2B were as follows: mucosal neuroma and Marfan-like habitus were observed in 99% of all cases, MTC was observed in 95%, and PHEO was observed in 50% [2, 13, 14]. However, more than 95% of the patients with MEN2B carried the p. M918T mutation of *RET*. After we searched Chinese articles in the Wanfang and CNKI databases, we found 20 MEN2B cases, of which 35% of the patients (7 patients) were diagnosed in childhood. Among these patients, 95, 95, 80, 50, and 50% had MTC, mucosal neuroma, Marfan-like habitus, PHEO, and GI symptoms, respectively. After thyroidectomy, most patients had high serum calcitonin levels. It is still unknown whether these patients are still alive. Among these patients, 12 had gene sequencing, and all of them had p.M918T mutations in *RET*.

At present, genetic testing is still the gold standard for diagnosing MEN2B. Because of the low incidence and poor prognosis of MEN2B, *RET* gene screening should be performed early for suspected cases.

MTC is a poorly differentiated cancer with a high degree of malignancy. Since MTC is not sensitive to radiotherapy and chemotherapy, surgery is the only cure for MTC. For MTC in the early stage without metastasis, the curative rate of thyroidectomy may reach 100%. Once lymph node metastasis occurs, however, the risk for metastasis after lymph node dissection is also high [15]. Targeted drugs such as vandetanib and cabozantinib have been used for metastatic MTC [16–18]. It was confirmed that these targeted drugs significantly alleviate clinical symptoms [17]_._ The use of these drugs is also warned against because of the potential side effects of heart disease. MTC may develop resistance to these drugs within a few years. Furthermore, the safety and efficacy of such drugs in children are unclear. In recent years, some new *RET* inhibitors, such as alectinib (Roche), Blu-667 and Loxo-292, have been tested in Phase I trials [19]. When the calcitonin doubling time was between 0.5 and 2 years, the 5-year and 10-year survival rates of MTC were 92 and 37%, respectively. Furthermore, when the calcitonin doubling time was < 6 months, the 5-year and 10-year survival rates decreased to 25 and 8%, respectively [20, 21]. In this case, her calcitonin doubling time was more than 6 months. In addition, she started puberty, had a growth spurt after her second operation, and menstruated when she was 14 years old. These findings indicated that the condition of the female adolescent deteriorated relatively slowly. Her calcitonin and CEA levels were very high, which indicated there was a metastasis of the MTC despite no positive findings from the CT scan. Because vandetanib is not approved in mainland China, we could only monitor her more frequently and thoroughly during the follow-up period.

In conclusion, p.M918T mutation of the *RET* gene accounted for more than 95% of all cases of MEN2B. Intractable constipation due to HD may be an early warning sign of MEN2B. Clinicians should follow these patients for a long time and pay attention to the presence of neck masses, mucosal neuroma or elevated blood pressure. *RET* gene sequencing can distinguish patients suffering from MEN2B from those suffering from other conditions in a timely manner to provide early treatment, which is the most important factor for changing the prognosis of MEN2B.

## Data Availability

The data and materials are available from the corresponding author (Yuan Xiao) on reasonable request.

## Abbreviations

MEN 2B: Multiple endocrine neoplasia type 2B
MTC: Medullary thyroid carcinoma
MEN: Multiple endocrine neoplasia
FMTC: Familial medullary thyroid carcinoma
GI: Gastrointestinal
PHEO: Pheochromocytoma
ACMG: American College of Medical Genetics and Genomics guidelines,
CEA: Carcinoembryonic antigen
ATA: American Thyroid Association
CT: Computed tomography
